# Phonon density of states for α-plutonium from density-functional theory

**DOI:** 10.1038/s41598-019-55343-z

**Published:** 2019-12-10

**Authors:** Per Söderlind, Lin H. Yang

**Affiliations:** Lawrence Livermore National Laboratory, Livermore, California, 94550 USA

**Keywords:** Materials science, Condensed-matter physics, Electronic properties and materials

## Abstract

The ground-state α phase of plutonium has an extraordinary 16-atom per cell, monoclinic crystal structure defined by 20 parameters, including the cell dimensions, not dictated by the symmetry. The electronic, magnetic, and elastic properties of this complicated material have been predicted in the past but here we compute its phonon spectra. Employing a density-functional-theory (DFT) model, that is fully relativistic and accounts for orbital–orbital coupling (orbital polarization, OP), we determine the phonon density of states of α-plutonium and find good agreement with inelastic x-ray scattering. The calculated specific heat also compares very favorably with experiment. An analysis of the partial atom-projected phonon spectra suggests that atom type 8, that is located in a more open space of the structure, dominates the intensity at very high phonon frequencies. This feature of the model is essential for a good agreement with the experimental spectra. The satisfactory comparison between theory and experiment for the phonons and specific heat suggests that the DFT (+OP) approach is appropriate and accurate for α-plutonium.

## Introduction

Progress is being made in understanding the complex physics of plutonium metal thanks to advanced experimental work and theoretical modeling^1^. The development of a comprehensive physical picture is hampered because plutonium is difficult to deal with due to its toxicity, radioactivity, and scarceness, and by strict regulatory restrictions. Theoretical work is likewise arduous because electronic and crystal structures are complex and without counterparts in the periodic table of the elements. Furthermore, the 5f electrons are dominant in plutonium and they tend to correlate in ways that are challenging to predict.

For two decades the electronic structure of plutonium has been explained by essentially two contrasting behaviors of the 5f electrons. On one hand, the 5f electrons are interpreted as substantially delocalized (band electrons) and within this model the counterintuitive and astounding ground-state α phase is understood^2^. On the other hand, the 5f electrons are postulated to be largely localized (atomic like) and this picture is used^3^ to explain the high-temperature δ phase of plutonium. For the last twenty years, many papers have been published focusing on either of these two opposing views for plutonium’s electronic structure, see for example ref. ^4–21^.

We argue that recent dynamical mean-field theory (DMFT) and related DFT + U calculations^22,23^ have helped to clarify the contrasting views on plutonium’s electronic structure outlined above and in ref. ^2,3^. The crux of the argument is that the strength of the electron correlations promoting the localization of the 5f electrons has been determined to be much weaker^22,23^ than previously assumed^3^. In terms of intra-atomic Coulomb interactions, the effective Hubbard U is claimed to be about 1 eV for all plutonium phases (and uranium as well)^22,23^ in contradiction to the conventionally expected large U ~ 4–4.5 eV^3^. A large effective U parameter in DFT + U-related approaches is a prerequisite for 5f-localization in plutonium. Contrarily, it was recently shown^24^ that the existence of a small Hubbard U (~1 eV) is more consistent with delocalized character of the 5f electrons, particularly when spin–orbit coupling and orbital–orbital interaction (orbital polarization) are considered. The only way to conclusively decide between these theoretical models for plutonium is to gather experimental evidence that support or contradict the respective approach. Lattice vibrations or phonons are fundamental properties that provide such evidence.

In our review paper^24^, we demonstrate that many of plutonium’s properties can be understood in terms of a DFT model and strong spin–orbit and orbital–orbital interactions within the delocalized 5f-band framework. Particularly, the model explains the chemical 5f-electron bonding that governs the phase diagram, lattice constants, elastic constants, and phonons (δ plutonium). For ε plutonium the phonons and elastic constants have also been predicted^20,21^ but judging their quality will await experimental studies. Theoretical lattice dynamics for the complex α phase has been avoided, however, because of technical and computational barriers that have made it too onerous to investigate. Actually, to our knowledge, no first-principles attempt to determine the lattice dynamics for α-plutonium has been reported. The reason is that the low-symmetry multi-atom monoclinic cell does not lend itself easily to phonon calculations. Of course, the 5f electrons also require special care, related to relativistic and magnetic interactions, that further complicates the situation.

Experimentally, the phonon density of states for α-plutonium was obtained from inelastic x-ray diffraction measurements a decade ago^25^. The accuracy of the spectra was corroborated by the fact that it was compatible with the measured heat capacity. In the present report, we introduce a first-principles-theory investigation of the lattice dynamics and heat capacity for α-plutonium.

## Results

In most of the earlier investigations of α-plutonium the details of the monoclinic structure were assumed to be those of Zachariasen and Ellinger^26^, observed at room temperature. Our DFT framework, however, considers zero temperature and a more thorough investigation of α-plutonium requires that the structure is resolved from theory. This was done recently^24^ and the theoretical structural parameters at zero temperature are actually very close to the room-temperature x-ray results^26^. The small differences (less than 2%), however, have a slight but noticeable effect on the chemical bonding and related properties of α-plutonium.

In Table 1 we compare previous results^16^ from calculations that adopt the experimental geometry with more recent computations^24^ where the structure is determined from theory. The later, more accurate, calculations result in a somewhat smaller atomic volume and larger bulk modulus. There is also a relatively small energy gain from the structural optimization (ΔE ~ 1 mRy/atom). At first glance, it appears that this new modeling is worsening the agreement with experiment, but one has to remember that theory predicts zero-temperature values while the measurements are recorded at room temperature^27–29^. With this in mind (thermal expansion is large in α-plutonium), the results from theoretically resolving the crystal structure in the latter calculation^24^ indicate an improved agreement with experiment.

**Table 1:** For α-plutonium, the atomic volume, V, and bulk modulus, B, for DFT calculations assuming the experimental^16^ or the optimized theoretical^24^ α structure. ΔE is the relative energy. Experimental data are from ref. ^27–29^.

Method	Structure	V (Å^3^)	B (GPa)	ΔE (mRy/atom)
DFT^16^	Experimental	20.3	50	1
DFT^24^	Theoretical	19.7	70	0
Expt	n/a	20.0–20.4	37–66	n/a

**Figure 1:**
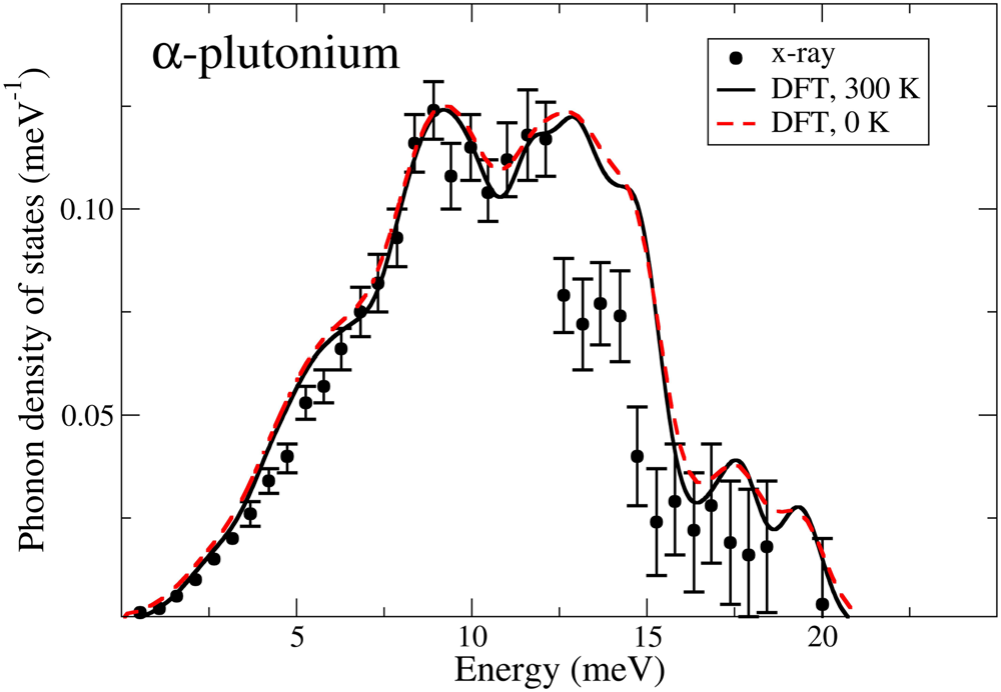
Present DFT and inelastic x-ray diffraction^25^ phonon density of states for α-plutonium. The solid line corresponds to a scaling that corrects for room temperature and the red dashed line the uncorrected (T = 0) result.

For α-plutonium, in its theoretical structure, we compute the lattice dynamics in the harmonic approximation by evaluating forces associated with finite displacements of the atoms in a supercell. The method is referred to as the small-displacement method^30^ and more details are provided in the Methods section below. In Fig. 1 we present the main result of this report, namely, our calculated α-plutonium phonon density of states together with results from inelastic x-ray diffraction^25^. As displayed in Table 1, the zero-temperature theory predicts (correctly) a smaller atomic volume than what is observed at room temperature. The difference can be corrected for by scaling the phonon density of states (quasi-harmonic approximation) and the result from this is also shown in Fig. 1. The effect of the thermal expansion is small, but it slightly improves the agreement with x-ray diffraction^25^, that is actually very good. Only in the narrow 12–13 meV region is there discrepancy that definitely lies outside of the experimental error bars. The rather encouraging comparison between our model and experiment may not be surprising, however, considering that the DFT model produces good elastic constants for α-plutonium^31^.

The Debye temperature, Θ_D_, is a quantity that is important for thermodynamical and equation-of-state modeling and it can be calculated directly from our phonon density of states. In this case, Θ_D_ = 161 K that should be compared to Wallace’s analysis (162 K)^32^, Debye-Grüneisen theory (167 K)^33^, and experiments (153 K)^34^. Apparently, there is a remarkable consistency in the Debye temperature between these methods for α-plutonium.

Furthermore, Manley *et al*.^25^. measured the specific heat at constant pressure (C_P_) for α-plutonium and related it to the specific heat at constant volume (C_V_). They evaluate the C_V_ from the measured energy dependence of the phonon density of states, dos(ε):

1 $${C}_{V}(T)=\frac{\partial }{\partial T}[{\int }_{0}^{\infty }\varepsilon dos(\varepsilon )n(\varepsilon ,T)d\varepsilon ].$$

Here, n(ε, T) is the Bose-Einstein thermal-occupation factor. For an appropriate comparison, the C_V_ needs to be converted to C_P_ and by adding terms related to thermal expansion and electronic contributions, one obtains:

2 $${C}_{P}(T)={C}_{V}(T)+9B\nu {\alpha }^{2}T+\gamma T.$$

Here, B is the bulk modulus, ν the specific volume, α the linear coefficient of thermal expansion, and γ represents the electron contribution. Numerical values for these material parameters were provided by Manley *et al*.^25^. The agreement between their calculated C_P_ (from the phonons) and the directly measured C_P_ was quite satisfactory. We can of course make the same comparison by evaluating C_V_ from Eq. (1) and our predicted DFT phonon density of states, and add the terms from Eq. (2) to derive C_P_. For this purpose, we are consistent with Manley *et al*.^25^. and use the same material parameters they do.

**Figure 2:**
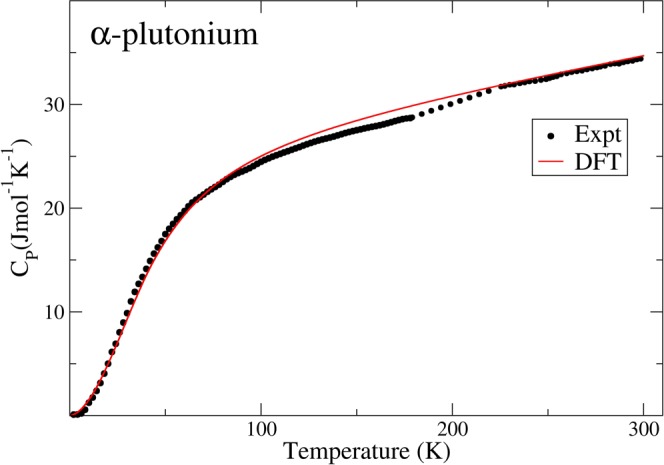
Present DFT and experimental^25^ specific heat at constant pressure, C_P_.

In Fig. 2 we show our theoretical specific heat at constant pressure, C_P_, together with the direct measurements^25^. For clarity, the error bars of the observed data are not included in the plot but can be found in the original report. However, the theoretical C_P_ values are safely within all these error bars and the agreement is very favorable, suggesting that our calculated phonon density of states is rather accurate as well.

## Discussion

**Figure 3:**
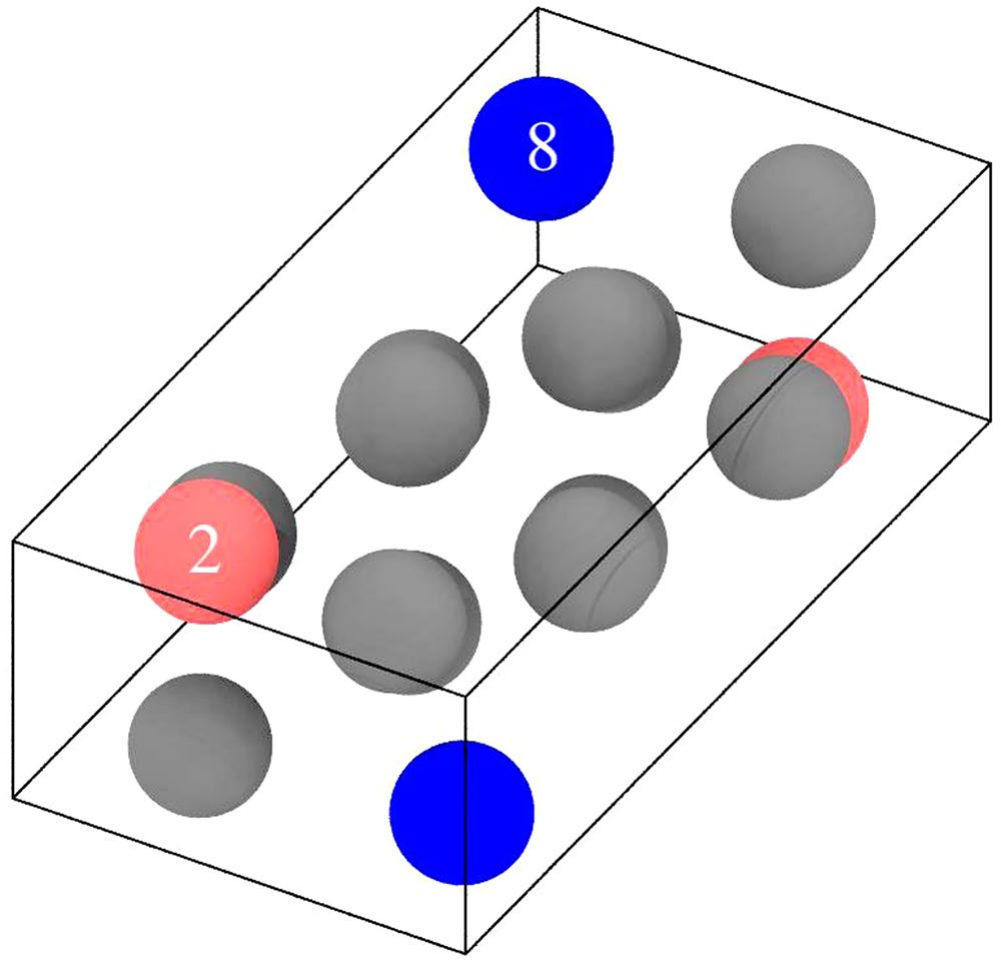
The α-plutonium monoclinic structure. Notice that atom type 8 (blue color) is positioned in a more open space than atom type 2, for example (red color).

**Figure 4:**
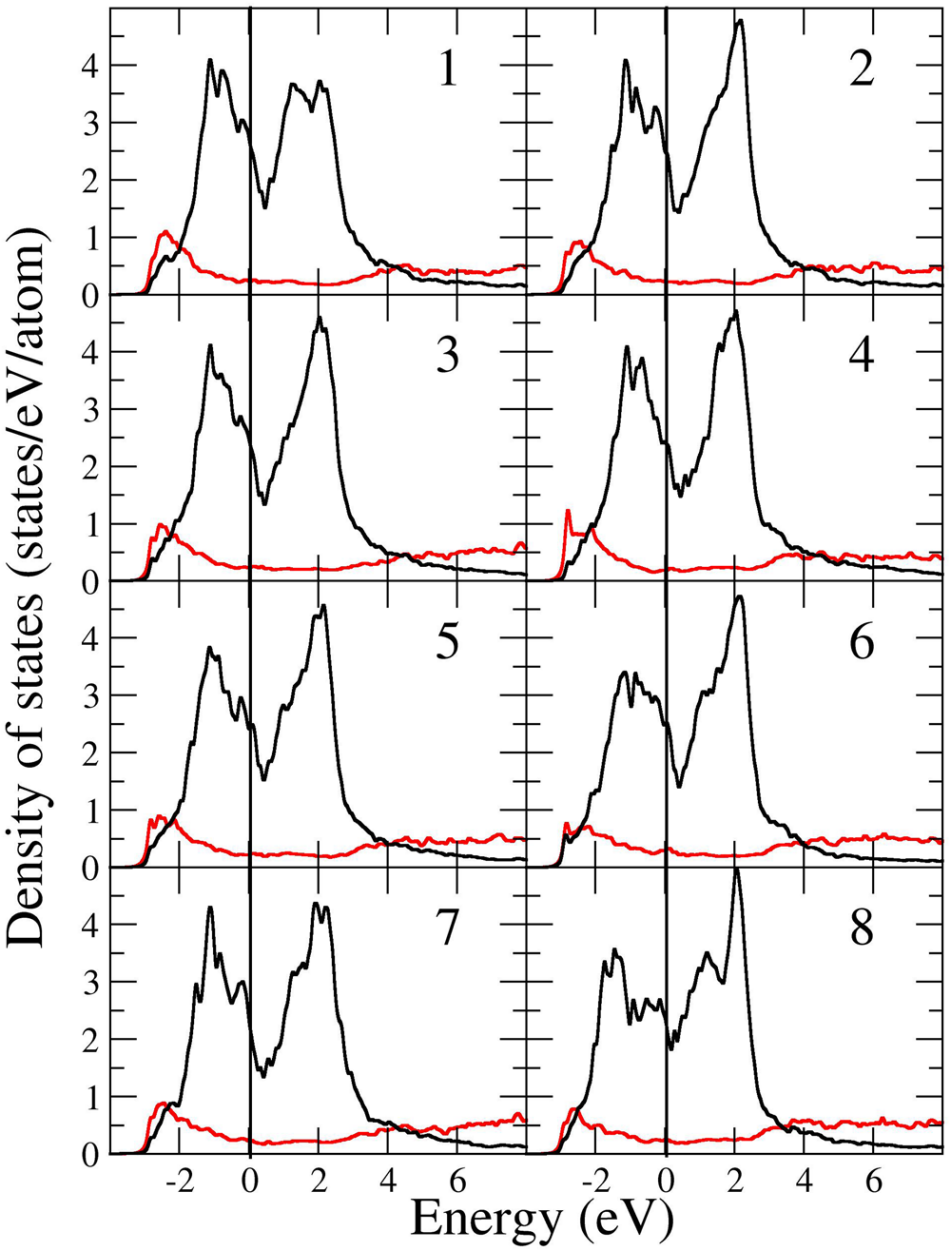
Calculated atom-projected (1–8 types) electronic density of states for α-plutonium. Black lines show 5f contributions and red lines show 6d contributions. Other contributions are negligible. The Fermi level is indicated with a vertical line at zero energy. Redrawn from ref. ^24^.

The monoclinic structure that defines the α phase of plutonium is quite remarkable. Not only is it without comparison, but the large monoclinic cell has sites that are very different from each other. Most glaring is the site for the 8^th^ atom (Zachariasen and Ellinger’s ordering^26^) that is located in a more open space than the other types, see Fig. 3. Consequently, the local electronic structure is significantly different between the atom types and particularly so for atom number 8. In Fig. 4 we show our calculated atom-projected electronic density of states^24^, and focusing on the features at the Fermi level, it is clear that this atom is different than all the others. One may hypothesize that the phonon properties are affected anomalously due to the contribution from this prominent 8^th^ atom.

**Figure 5:**
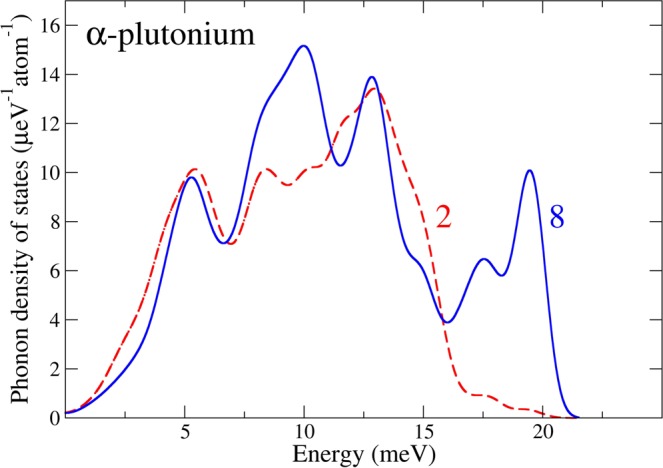
Calculated local atom-projected phonon density of states for atom types 2 (red-dashed) and 8 (blue-solid).

In Fig. 5 we plot the local atom-projected phonon density of states for atom types 2 and 8. We recognize immediately that type 8 has high-energy vibrational states that are completely absent for type 2. None of the other atom types have similar intensity at high energies (not shown). Therefore, atom 8 is mostly responsible for the 18–20 meV region of the phonon density of states for α-plutonium. Interestingly, this high-energy feature is very different from the phonon density of states of δ-plutonium (face-centered cubic) that has no intensity at all above about 15 meV^25^. It is apparent that good phonon properties of α-plutonium can only be modeled by a theory that correctly captures the unusual behavior of the special atom number 8.

## Methods

We divide the Methods section into two parts, because our calculations naturally fall into separate categories.

First, we focus on the density-functional theory and the electronic structure that is employed for calculating forces on displaced atoms. The electronic structure is constructed from a full-potential linear muffin-tin orbitals method^35^ that is fully relativistic but also includes an extension with an orbital-polarization contribution^36^. It is derived from atomic physics that is known to be an important perturbation of the 5f-electron states of plutonium^24,37^. The generalized gradient approximation is assumed for the electron exchange and correlation interactions. No parameters are adjustable; the (Racah) parameters associated with the orbital polarization are calculated self-consistently from Slater integrals of wave functions as described before^24,36^.

In our electronic-structure model for α-plutonium we consider, as in previous studies^24^, anti-ferromagnetic ordering with small net magnetic moments on the plutonium atoms (on average 0.15 μ_B_) due to the effective compensation between spin and orbital contributions. Spin fluctuations^38^ or non-collinear spins are in principle not ruled out in this theory, but due to computing-resources boundaries, we limit ourselves to the anti-ferromagnetic static-spin regime.

For the small-displacement method, forces on atoms that experience small displacements from their equilibrium positions need to be known. Here, we compute these atomic forces directly from the total-energy response to very small movements (±~ 0.01 Å) of each atom (4 displacements each for the three coordinates). A 4^th^-degree polynomial is then adapted to the energies and the force component is extracted from an analytical differentiation. Hence, there is no need to compute Hellman-Feynman forces that are troublesome to determine accurately and also not easily defined when substantial spin–orbit interaction and orbital polarization are present. This total-energy-derived forces scheme was initially employed for studying phonons in relativistic γ-uranium^39^ and has proven to be both robust and accurate in many applications since then.

The supercell required for the small-displacement method consists of 32 atoms (1 × 1 × 2) per cell and a total of 12 k points are used in integrations over the Brillouin zone. The energy eigenvalues are broadened with a Gaussian with 20 mRy energy width. Most other computational details are the same as in our recent review article on plutonium^24^.

The phonon density of states and thermal properties are accessed by the small-displacement approach implemented in the PHONOPY code^40^. This computational package solves the phonon equations in the harmonic approximation for a constant atomic volume. By adjusting the volume to account for thermal expansion (as we have done in Fig. 1) one rather applies a quasi-harmonic approximation of the phonons. The volume adjustment (scaling) is straightforward. The phonon density of states, dos(ε), is related to the atomic volume (V) and number (N) of occupied vibrational energy levels (ε_i_) so that

3 $$dos(\varepsilon )=\,\frac{1}{V}\mathop{\sum }\limits_{i=1}^{N}\delta (\varepsilon -{\varepsilon }_{i}),$$

where, δ(ε) is Dirac’s delta function. To scale the phonons to room temperature we account for the thermal expansion and replace V in Eq. (3) with the experimental room-temperature atomic volume for α-plutonium (20.0 Å^3^).

From the DFT total-energy-derived forces and PHONOPY we obtain real-space force constants. The dynamical matrices are then constructed using the force constants and phonon frequencies or other thermodynamical properties such as the heat capacity can readily be obtained. A total of 32 different supercells are required to determine the complete set of force constants necessary for computing the phonon frequencies in this fashion. In addition, the optimized α-plutonium structure is close but not in exact equilibrium and consequently some atoms have small residual forces (<0.1 eV/Å). These residual forces are subtracted off when establishing the force constants. This subtraction, however, did not change the phonons significantly. Finally, the phonon density of states, in Figs. 1 and 5, are broadened with a Gaussian function with an energy-width of 0.5 meV.
